# Studies on the Increasing Saltiness and Antioxidant Effects of Peanut Protein Maillard Reaction Products

**DOI:** 10.3390/antiox13060665

**Published:** 2024-05-29

**Authors:** Wenjing Xing, Chunmin Ma, Yang Yu, Fenglian Chen, Chunhua Yang, Na Zhang

**Affiliations:** College of Food Science and Engineering, Harbin University of Commerce, Harbin 150028, China; xingwenjing0219@163.com (W.X.); chunmin_ma@163.com (C.M.); yyhsd2024@163.com (Y.Y.); finesxm@163.com (F.C.)

**Keywords:** peanut protein, Maillard reaction, saltiness, antioxidant capacity

## Abstract

The salt taste-enhancing and antioxidant effect of the Maillard reaction on peanut protein hydrolysates (PPH) was explored. The multi-spectroscopic and sensory analysis results showed that the Maillard reaction products (MRPs) of hexose (glucose and galactose) had slower reaction rates than those of pentose (xylose and arabinose), but stronger umami and increasing saltiness effects. The Maillard reaction can improve the flavor of PPH, and the galactose-Maillard reaction product (Ga-MRP) has the best umami and salinity-enhancing effects. The measured molecular weight of Ga-MRP were all below 3000 Da, among which the molecular weights between 500–3000 Da accounted for 46.7%. The products produced during the Maillard reaction process resulted in a decrease in brightness and an increase in red value of Ga-MRP. The amino acid analysis results revealed that compared with PPH, the content of salty and umami amino acids in Ga-MRPs decreased, but their proportion in total free amino acids increased, and the content of bitter amino acids decreased. In addition, the Maillard reaction enhances the reducing ability, DPPH radical scavenging ability, and Fe^2+^ chelating ability of PPH. Therefore, the Maillard reaction product of peanut protein can be expected to be used as a substitute for salt seasoning, with excellent antioxidant properties.

## 1. Introduction

Peanut is one of the most important oil crops in the world, containing fats, proteins, vitamins, minerals, and other nutrients. The global annual production of peanuts is about 45 million tons, and the protein content of peanuts is generally 25% to 30% [1]. Peanut protein (PP) contains eight essential amino acids, especially aspartic acid and glutamic acid, and its nutritional value is similar to animal protein. In addition, compared to soy protein with higher utilization, peanut protein lacks soy flavor, has fewer anti-nutritional factors, and has a higher digestibility rate of over 90%, which makes it easier to be absorbed. Numerous studies have demonstrated that peanut protein has good functional activities after enzymatic digestion, fermentation, glycosylation, and other processes, such as blood pressure-lowering [2], antioxidant [3], and antimicrobial activities [4], as well as chelating metal ions [5]. For example, Li et al. [6] modified peanut protein by glycosylation and compared its structure and solubility with that of natural peanut protein, and found that the solubility of protein–polysaccharide complexes was significantly improved. Jamdar et al. [7] found that the ferrous ion chelating activity, DPPH scavenging activity, and ACE inhibitory activity of peanut protein hydrolysates (PPH) increase with the degree of peanut protein hydrolysis. In addition, peanut proteins are also known to produce flavor compounds after the Maillard reaction. In a study, the hydrolysate of peanut protein isolate showed a stronger umami enhancement effect after the Maillard reaction with glucose [8]. It can be seen that peanut protein has the potential to develop products with flavor and functional properties; however, in actual production, peanut meal is mostly used as animal feed or fertilizer, and the utilization rate of peanut protein is low. Given that peanut meal has the advantages of sufficient materials and being rich in proteins, it is of great significance to develop and utilize peanut protein resources to improve the comprehensive utilization value of peanuts.

High sodium intake is now a major dietary risk factor for non-communicable diseases worldwide. Excessive sodium intake increases the risk of hypertension, which in turn increases the risk of cardiovascular disease, stroke, and other serious illnesses. Due to dietary habits and the influence of the food processing industry, the salt intake of most people far exceeds the recommended daily salt intake of 5 g for adults by the World Health Organization. Sodium chloride (NaCl) is the main chemical component of salt, and 90% of dietary sodium intake exists in the form of NaCl. Excessive intake of sodium in the daily diet may lead to a range of health problems, such as high blood pressure, coronary heart disease, and kidney disease [9]. Therefore, reducing sodium intake without reducing the saltiness of foods is crucial. Researchers have proposed a variety of strategies to reduce sodium content while maintaining the perceived saltiness of processed foods, including directly reducing salt content [10], altering the physical form of salt [11], and using salt substitutes [12]. The direct purpose of the reduction of salt content is to reduce salt intake without affecting people’s perception of food saltiness, and this method requires a long time. Changing the physical form of salt is mainly conducted to optimize the size and shape of salt, or change the spatial distribution of salt, which is mainly used in bakery products such as bread; however, the scope of application is relatively limited. Salt substitutes mainly include non-sodium salts or salty peptides obtained through enzymatic technology, etc. Replacing part of the salt with other salty or increasing saltiness compounds to enhance the perception of salty taste has a wider scope of application. At present, the most widely used non-sodium salt is mainly potassium chloride, but research has found that when more than 30% of NaCl is replaced by KCl, it will lead to the production of a bitter and metallic taste, which is difficult to be widely accepted by consumers [13]. Enzymatic hydrolysis of proteins to obtain enzyme products can increase food salinity, but it also has the disadvantage of producing bitterness [14]. By contrast, improving the taste of proteolytic products through the Maillard reaction can enhance both saltiness and umami, while reducing the bitterness [15]. Therefore, Maillard products have great potential in the field of salt-reduced seasoning. However, the mechanism by which the products of the Maillard reaction increase salinity is still unclear.

Maillard reaction refers to the carbonyl ammonia reaction between the carbonyl group of reducing sugars and the amino group of amino acids, which can not only give the color and flavor quality of food but also improve the functional properties of protein hydrolysate. For example, Liu et al. [16] found that enzymatic hydrolysis and glycosylation can destroy the globular structure of ovalbumin, forming a tightly ordered reticulation structure, which increases the contact area with water and oil and thus improves the emulsification of proteins. In addition, a large number of studies have found that the Maillard reaction product has strong reducing ability [17], DPPH and ABTS radical scavenging ability [18,19], and inhibition of lipid peroxidation [20]. Naik et al. [21] analyzed the products of amaranth-red seaweed coupling after the Maillard reaction and found that the Maillard reaction significantly enhanced the solubility, emulsification, and antioxidant properties of the proteins. Viturat et al. [22] prepared chitosan-based nanoparticles with enhanced antioxidant activity by an ultrasound-assisted Maillard reaction of chitosan and glucose. Han et al. [23] prepared the Maillard reaction product by reacting scallop hydrolysate with ribose. They found that the Maillard reaction can potentially be used as a food antioxidant to inhibit lipid oxidation or protect cells from oxidative damage.

This study, therefore, aims to prepare modified products with salt-enhancing properties through the peanut protein Maillard reaction. Four Maillard reaction products were prepared by hydrolyzing peanut protein with two enzyme complexes (papain and flavourzyme) and introducing four reducing sugars (xylose, glucose, arabinose, and galactose). The distribution of molecular weight and color difference were measured to determine the generation of reaction products. The salinization effects of xylose-MRPs, glucose-MRPs, arabinose-MRPs, and galactose-MRPs were compared through electronic tongue and sensory evaluation. The influence of different reducing sugar structures on the structural characteristics of Maillard reaction products was analyzed through UV and infrared spectroscopy. The changes in free amino acid content before and after the Maillard reaction were analyzed, and the reasons for the increase in salinity of MRPs were analyzed. In addition, the antioxidant properties of PPH and MRPs were studied to reflect the potential of peanut protein Maillard reactants as food antioxidants. This study provides a reference for the development of foods with salt reducing and antioxidant effects.

## 2. Materials and Methods

### 2.1. Materials

Peanut protein (750.0 g/kg, dry basis) was purchased from Xi’an Pnostic Bio-environmental Technology Co., Ltd. (Xi’an, China). Flavourzyme (150 kU/g) and papain (800 kU/g) was obtained from Shanghai Macklin Biochemical Co., Ltd. (Shanghai, China). L-arabinose, D-glucose, D-galactose, D-xylose, and L-cysteine (food grade) were bought from He’nan Wanbang Chemical Technology Co., Ltd. (Zhengzhou, China). 1,2-Dichlorobenzene standard solution and phenanthrozine were purchased from Shanghai Aladdin Biochemical Technology Co., Ltd. (Shanghai, China). Potassium ferricyanide, ferric chloride, ferrous chloride, trichloroacetic acid, and sodium chloride were all brought from Shanghai Yuanye Bio-Technology Co., Ltd. (Shanghai, China). Other reagents were of analytical grade, while all solutions were prepared in distilled water and were fresh before using.

### 2.2. Preparation of Enzyme-Hydrolyzed Peanut Protein and Its Maillard Reaction Products

The preparation of PPH was carried out according to our previous enzymatic hydrolysis conditions [24]. Briefly, peanut protein powder was mixed with distilled water in a ratio of 1:10 (*w*/*v*) and heat-treated at 95 °C for 20 min. The pH value of the initial solution was adjusted to 6.5 with 2 mol/L sodium hydroxide, and then papain (3020 U/g) was added and stirred at 55 °C using a magnetic stirrer with constant temperature heating (DF-101S, Yuhua, Zhengzhou, China) for 2.8 h. Then, flavourzyme (610 U/g) was added and continuously stirred for 3.8 h. After the reaction was completed, the mixed solution was heated in boiling water for 10 min to inactivate the enzyme and terminate enzyme hydrolysis. After cooling, the mixed solution was centrifuged at 4000 rpm for 40 min using a low-temperature, high-speed centrifuge (H1650R, Xiang Yi Laboratory Instrument, Changsha, China). The supernatant was collected and freeze-dried using a freeze dryer (SCIENTZ-12N, Ningbo, China) to obtain PPH with a hydrolysis degree of 16.65% (obtained from previous study) [24].

The preparation of MRPs was improved on the study of Sun et al. [25]. PPH (4 g) was mixed with 1.2 g of four small-molecule sugars (glucose, galactose, xylose, and arabinose), and then deionized water was added to achieve a final protein concentration of 10%. The pH value of the solution was adjusted to 7.5 with 1 mol/L NaOH. The solution was subjected to a Maillard reaction in an oil bath (DXY-2H, DingXingyi, Shenzhen, China) using a temperature of 120 °C and maintained in the oil bath for 2 h. The mixture was transferred to an ice bath and cooled to stop the Maillard reaction. The reactant was centrifuged at 10,000 rpm for 20 min, and the supernatant was collected. The samples were freeze-dried and stored at 4 °C for use. The samples were named glucose-MRP (Gl-MRP), galactose MRP (Ga-MRP), xylose-MRP (X-MRP), and arabinose-MRP (A-MRP), respectively.

### 2.3. Spectral Determination of Maillard Reaction Products of Peanut Protein

#### 2.3.1. Ultraviolet Absorption Spectra Assay

Ultraviolet absorption spectra of PP, PPH, and four MRPs (Gl-MRP, Ga-MRP, X-MRP, and A-MRP) were found according to the method of Wang et al. [26]. The protein concentration of the samples was diluted to 1 g/L with phosphate buffer solution (pH 7.0, concentration 0.01 mol/L), and then the samples were detected using a UV–visible spectrophotometer (T6 New Century, Pukin Instruments, Beijing, China). The samples were scanned at a sampling interval of 1 nm, with a wavelength scanning range of 190–400 nm. The absorbance of the samples was determined and compared for the four MRPs (X-MRP, Gl-MRP, A-MRP, and Ga-MRP), PP, and PPH.

#### 2.3.2. Fourier Transform Infrared Spectroscopy (FT-IR) Assay

The FTIR spectra of PP, PPH, and four MRPs (Gl-MRP, Ga-MRP, X-MRP, and A-MRP) were determined according to the method of Liu et al. [27]. The samples were mixed with potassium bromide at a mass ratio of 1:100, and pressed into a transparent sheet. The samples were scanned in the 4000–400 cm^−1^ region using an infrared spectrometer (PerkinElmer, Waltham, MA, USA). The resolution was set to 4 cm^−1^, and each sample was measured three times to analyze their molecular structure.

#### 2.3.3. Fluorescence Spectroscopy Assay

The fluorescence spectra of PP, PPH, and four MRPs (Gl-MRP, Ga-MRP, X-MRP, and A-MRP) were determined according to the method of Liu et al. [28] with slightly modifications. The fluorescence spectra were scanned at room temperature using a fluorescence spectrophotometer (Lumina. Thermo Fisher Scientific, Shanghai, China). The samples tested were fixed at a concentration of 1 g/L using a phosphate buffer with a pH of 7.0. The excitation wavelength was set at 347 nm, while the emission wavelength was 370~550 nm. The phosphate buffer was used as the blank control.

### 2.4. Sensory Properties of Maillard Reaction Products of Peanut Protein

#### 2.4.1. Electronic Tongue Assay

The sensory properties of PP, PPH, and four MRPs (Gl-MRP, Ga-MRP, X-MRP, A-MRP) were determined using an electronic tongue sensor system (TS-5000Z, INSENT, Tokyo, Japan), referring to the research method of He et al. [29]. The MRPs (Gl-MRP, Ga-MRP, X-MRP, A-MRP) of 0.5% (*w*/*w*) was mixed with 0.5% (*w*/*w*) NaCl solution to prepare the sample solution. The sample solution (40 mL) was accurately pipetted into a special measuring cup, and was measured using five sensors, including sourness, bitterness, saltiness, umami, and astringency in the flavor analyzing system. Each sample was measured four times, and the sample testing time was 120 s. The instrument was stabilized after the first measurement, and the average value of the signal data of the last three times was taken as the taste signal intensity of the sample.

#### 2.4.2. Sensory Evaluation Assay

A sensory evaluation panel consisting of 10 internal reviewers (five females and five males, aged 20–30 years old) was established in order to perform a sensory evaluation according to the method of Yu et al. [30], with slight modifications. The sensory evaluation was conducted in a sensory analysis laboratory by tasting 0.5% (*w*/*w*) PPH, X-MRP, Gl-MRP, A-MRP, and Ga-MRP solutions dissolved in 0.5% (*w*/*w*) NaCl solution. The umami, salty, and bitter tastes were evaluated using 0.5% (*w*/*w*) NaCl solution as the reference standard. A 10-point scale was used, in which the sensory intensity of the distilled water was at 0 points and the sensory intensity of the reference standard solution was at 5 points. The evaluation was repeated three times for each sample and the average value was taken as the final score.

### 2.5. Color Difference Assay

The color change of the samples (PP, PPH, and Ga-MRP solutions) was carried out by referring to the method of Zha et al. [31], using a portable computerized colorimeter (NR200, 3nh, Shenzhen, China). The instrument was calibrated with a standard whiteboard. The sample solutions were poured into a transparent beaker to make sure that the liquid surface was flat and free of air bubbles. Then, the samples were placed into the transmittance measurement port of the instrument, and the color characteristics of the samples were determined. Each sample was measured three times. The L*, a*, and b* values of the samples were recorded, where L* represented the luminance value, a* represented the redness value, and b* represented the yellowness value.

### 2.6. Molecular Weight Distribution Assay

The molecular weight distribution of the Ga-MRP was detected according to the method of Wu et al. [32] using a high-performance liquid chromatograph (Waters 2695, Milford, MA, USA) equipped with a TSK GMPW XL column (300 mm × 7.8 mm, Tosoh Corp., Tokyo, Japan). The column temperature was set as 30 °C, flow rate was 0.5 mL/min, and detection wavelength was 220 nm. The mobile phase was the mixture of acetonitrile, water, and trifluoroacetic acid at a volume ratio of 40:60:0.1. Cytochrome C (12,500 Da), protease-inhibiting peptide (6500 Da), bacillus peptide (1450 Da), tetrapeptide GGYR (451 Da), and tripeptide GGG (189 Da) were used as standard solutions. The results were processed by the self-contained GPC software (Empower 3) of the chromatograph, and the relative molecular mass of the peptides and their distribution in the samples were calculated by the standard curve equation.

### 2.7. Determination of Amino Acids Contents

The determination of amino acid content was carried out according to the method of Zhao et al. [33] with slight modifications. Pre-treatment for total amino acid determination was as follows. A volume of 8 mL HCl (6 mol/L) was added to the freeze-dried sample (100 mg), and nitrogen was added to keep the solution slightly boiling. The lid of the hydrolysis tube was tightened, and the sample was hydrolyzed at 110 °C for 24 h. An NaOH neutralization solution (10 mol/L) of 4.8 mL was added, and then the volume was set to 25 mL with deionized water. The solution was filtered with double-layer filter paper and centrifuged at 10,000 rpm for 10 min. The supernatant was collected in a liquid-phase injection bottle and analyzed using an amino acid analyzer (S433D, SYKAM, Munich, Germany).

Pre-treatment for free amino acid determination was as follows. The sample (100 mg) was added to a 10% trichloroacetic acid solution of 1 mL, and allowed to stand at 4 °C for 2 h. The samples were then centrifuged at 10,000 rpm for 10 min at 4 °C, and 400 μL of the supernatant was collected in a liquid-phase injection vial, and analyzed by the amino acid analyzer.

An ODS Hypersil column (250 mm × 4.6 mm × 5 μm) was used for chromatographic analysis; mobile phase A was 0.6 mmol/L sodium acetate, while mobile phase B was 0.15 mmol/L sodium acetate/methanol/acetonitrile (at a volume ratio of 1:2:2). The flow rate was 1.0 mL/min, the column temperature was 40 °C, and the injection volume was set as 10 μL. The detection wavelength of the UV detector was 338 nm. A standard curve was prepared with standard amino acid mixtures and the amino acids were quantified using the external standard method.

### 2.8. Antioxidant Activity Assay

#### 2.8.1. Reducing Capability

The reducing power of PPH and Ga-MRPs was evaluated by referring to the method of Sampath et al. [34], with slight modifications. PPH sample solutions were diluted to different concentrations (10, 20, 30, 40, 50, and 60 g/L) and MRP sample solutions were diluted to different concentrations (1, 2, 4, 6, 8, 10, 12, and 14 g/L), and then 2 mL of 0.2 mol/L phosphate buffer (pH 6.6) and 2 mL of 1% potassium ferricyanide solution were added and mixed, respectively. The mixture solutions were reacted in a water bath at 50 °C for 20 min, cooled down, and 2 mL of 10% trichloroacetic acid solution was added. The mixed solution was centrifuged at 6000 rpm for 10 min using a high-speed centrifuge (H1650R, Xiang Yi Laboratory Instrument, Changsha, China). The supernatant (2 mL) was mixed with 2 mL of deionized water and 0.4 mL of 0.1% FeCl_3_. The absorbance of mixed samples at 700 nm was measured by a UV–visible spectrophotometer (T6 New Century, Pukin Instruments, Beijing, China) to reflect reducing capability.

#### 2.8.2. DPPH Radical Scavenging Activity

The DPPH radical scavenging capacity of PPH and Ga-MRPs was determined using the method of Liu et al. [35], with slight modification. PPH sample solution was diluted to different concentrations (10, 12, 15, 20, 25, 30, and 40 g/L) and MRP sample solution was diluted to different concentrations (0.05, 0.1, 0.2, 0.3, 0.4, 0.6, 0.8, 1.0, and 1.2 g/L). A solution of 2 mL was added to 4 mL of 0.1 mmol/L DPPH ethanol solution. The mixture was placed in a water bath at 33 °C for 30 min to react. The absorbance of the supernatant was determined at 517 nm, and the scavenging rate was calculated by the following formula:

DPPH radical scavenging activity (%) = [1 − (As − Ac)/Ab] × 100(1)

Abbreviations: As, the absorbance of the sample; Ac, the absorbance of the control (ethanol instead of DPPH ethanol solution); Ab, the absorbance of the blank (deionized water instead of sample solution).

#### 2.8.3. Fe^2+^ Chelating Ability

The Fe^2+^ chelating ability of PPH and the Ga-MRPs was evaluated by referring to the method of Pino et al. [36], with slight modifications. Sample solutions (1 mL) with different concentrations (0.5, 1.0, 2.0, 4.0, 6.0, 8.0, and 10.0 g/L) were mixed with 3.7 mL of deionized water and 0.1 mL of 2 mmol/L FeCl_2_ solution, and the mixture was left at room temperature for 3 min. Ferrozine solution (5 mmol/L) of 0.2 mL was added to the mixture to react at room temperature for 10 min. The absorbance at 562 nm was measured, and the chelation rate was calculated as follows:

Fe^2+^ chelation rate (%) = [(A_sample_ − A_control_)/A_blank_] × 100 (2)

A_sample_ represents the absorbance value of the mixture of test sample and ferrous chloride and iron zinc at 562 nm; A_control_ represents the absorbance value of a mixture of deionized water, ferrous chloride, and iron zinc at 562 nm; A_blank_ represents the absorbance value of the mixture of the test sample, ferrous chloride, and deionized water at 562 nm.

### 2.9. Statistical Analysis

The experiment was repeated three times, the graphs were scanned three times, and the data in the table represent the mean of the three replicates, and the results are expressed as mean or mean ± standard deviation. The experimental data were analyzed by ANOVA and significant differences were analyzed using SPSS 26.0 (SPSS Inc., Chicago, IL, USA). One-way ANOVA was used to analyze the significance with a threshold of *p*-value = 0.05. Graphs were plotted using Origin 2018 (Origin Lab Co., Northampton, MA, USA).

## 3. Results

### 3.1. Secondary Conformation Changes of PPH and Its Four Glycation Products

#### 3.1.1. UV–Visible Spectroscopy Analysis

**Figure 1:**
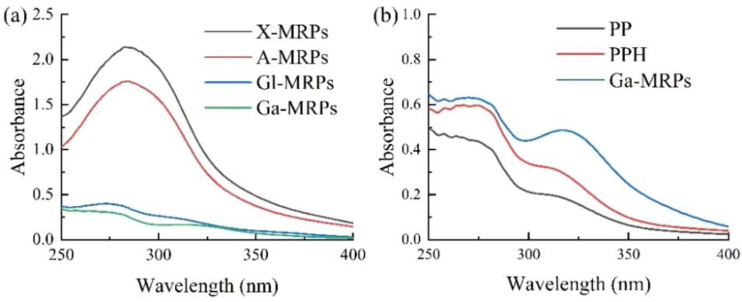
(**a**): UV absorption spectra of the Maillard reaction products with different reducing sugars; (**b**): UV absorption spectra of PP, PPH, and Ga-MRPs.

The generation of brown substances during the Maillard reaction can lead to changes in the UV absorption spectrum [37]. The UV absorption spectra of X-MRPs, A-MRPs, Gl-MRPs, and Ga-MRPs in the wavelength range of 250–400 nm are shown in Figure 1a. It can be seen that the absorption peaks of pentose MRPs (X-MRPs and A-MRPs) were significantly higher than those of hexose MRPs (Gl-MRPs and Ga-MRPs). This is because the structure of the sugar directly affects the rate of Maillard reaction, thereby affecting the formation of brown components in the Maillard reaction process. The UV absorption spectra of PP, PPH, and MRPs are shown in Figure 1b. The UV spectral absorption values of MRPs were higher than the absorption peaks of PP and PPH, which may be because the Maillard reaction produces intermediate and terminal products, increasing the amount of chromophore substances with UV absorption ability. PPH has a higher absorbance than PP, which may be due to the different molecular weights of small molecules obtained after enzymatic hydrolysis of PP, exposing the chromogenic groups and resulting in a significant increase in absorbance [38].

#### 3.1.2. FT-IR Spectroscopy Analysis

**Figure 2:**
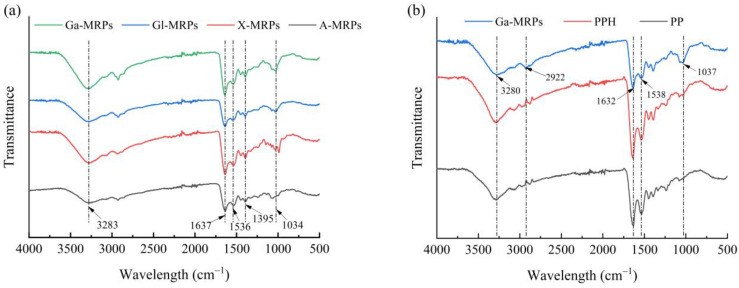
(**a**): FT-IR spectroscopy of the Maillard reaction products with different reducing sugars; (**b**): FT-IR spectroscopy of PP, PPH, and Ga-MRPs.

Fourier transform infrared (FT-IR) spectroscopy refers to the absorption of electromagnetic radiation energy at a certain frequency by a sample on the interferometer optical path, causing the vibration and deflection of some molecular groups. It is commonly used to identify functional groups and chemical structures and analyze the chemical composition of complex polymers and the secondary structure of proteins or peptides [20]. The Maillard reaction leads to the consumption of certain functional groups (such as -NH_2_) and the production of new functional groups such as C=O, Schiff base C=N, and pyrazine C-N in Amadori rearrangement products, resulting in changes in the infrared spectrum [39]. The infrared spectra of X-MRPs, A-MRPs, Gl MRPs, and Ga MRPs are shown in Figure 2a. There are no observed changes in the functional groups of MRPs in the FT-IR spectra, which may be due to the same reaction mechanism of sugar with proteins, all of which are the condensation of carbonyl and amino groups. However, the reaction rate or degree between different sugars and protein hydrolysates varies, so there is a difference in the vibration amplitude in the FT-IR. The absorption peak of the Maillard reaction product in the range of 3000–3500 cm^−1^ may be caused by the addition of hydroxyl groups and the consumption of amino groups, while the absorption peak at 1634 cm^−1^ is mainly due to the bending vibration of N-H bonds. The infrared spectra of PP, PPH, and MRPs (Figure 2b) indicated that compared to PP, the FT-IR spectrum of PPH not only did not produce new absorption peaks, but also did not remove the existing absorption peaks, suggesting that there was no change in the type of peanut protein products after enzymatic hydrolysis. The peak of Ga-MRPs at 3280 cm^−1^ reflects the stretching vibration of hydroxyl (-OH) functional groups under tensile stress. The peak at 1632 cm^−1^ is caused by the stretching vibration of the amide I band C=O. The absorption peak intensity of MRPs at 1037 cm^−1^ is significantly enhanced compared to that of PPH, and a blue shift phenomenon appeared, indicating that the introduction of reducing sugars onto PPH would change the structure of PPH, causing the vibration of the reactant side chains.

#### 3.1.3. Fluorescence Spectroscopy Analysis

**Figure 3:**
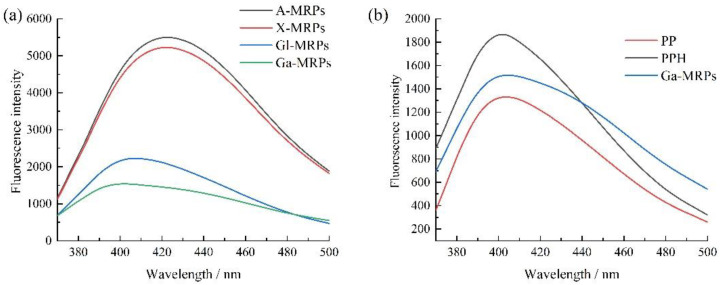
(**a**): Fluorescence spectra of the Maillard reaction products with different reducing sugars; (**b**): fluorescence spectra of PP, PPH, and Ga-MRPs.

The changes in fluorescence spectra are attributed to the microenvironment changes of tryptophan, tyrosine, and phenylalanine residues in proteins; therefore, fluorescence spectra can be used to characterize the structure of proteins [40]. The results showed that the fluorescence intensity of the pentose-modified MRPs was higher than that of the hexose modified MRPs. The order of fluorescence intensity from high to low was X-MRPs, A-MRPs, Gl-MRPs, and Ga-MRPs (Figure 3a). This is due to the chemical interaction between the carbonyl groups of sugar molecules and the amino groups of amino acid molecules in the Maillard reaction, resulting in different reaction rates due to the different structures of reducing sugars. Samples with faster reaction rates generate higher fluorescence intensity for MRPs. The fluorescence spectra of PP, PPH, and MRPs are compared in Figure 3b. It was found that the fluorescence intensity of PPH was higher than that of PP, which is because enzymatic hydrolysis caused peptide bond cleavage in peanut protein, producing some free amino acids and small molecule peptide segments, exposing tryptophan, tyrosine, and phenylalanine residues, and increasing fluorescence intensity. The fluorescence intensity of PPH is higher than that of MRPs, which may be due to the covalent binding between sugar molecules and peptides during the Maillard reaction, which shields the internal fluorescent groups.

### 3.2. Analysis of Sensory Properties of Four Maillard Reaction Products

#### 3.2.1. Sensory Evaluation of Four Maillard Reaction Products

**Figure 4:**
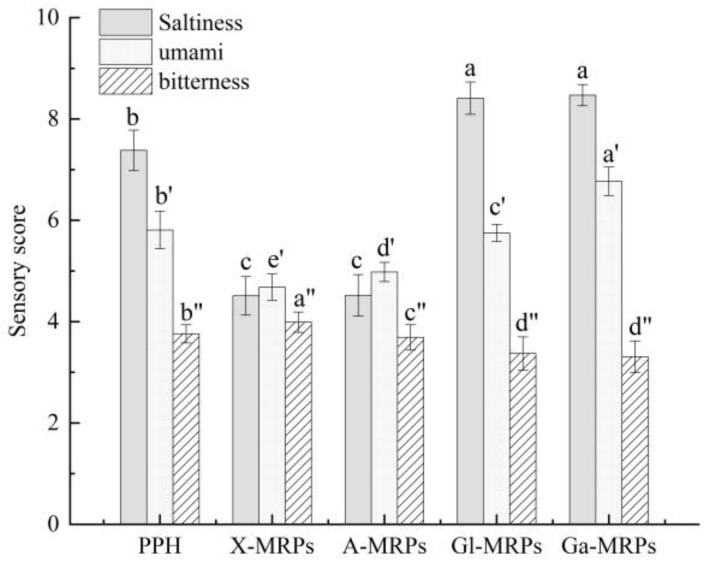
Sensory evaluation of PPH and four Maillard reaction products. Different lowercase letters (a–c, a’–e’ and a”–d”) above the columns indicate that one-way ANOVA of the mean values differs significantly (*p* < 0.05).

The sensory evaluation of four Maillard reaction products are shown in Figure 4. The solution of Ga-MRPs had the highest sensory evaluation values of 8.47 and 6.77 for saltiness and umami taste, respectively, while the lowest value of 3.31 for bitter taste. The hexose-Maillard products (Ga-MRPs and Gl-MRPs) had the higher saltiness and umami and the lower bitterness than PPH; however, the pentose-Maillard products (X-MRPs and A-MRPs) had significantly lower saltiness and umami taste compared to PPH. Therefore, the sensory properties of the products were related to the type of sugar, and the taste characteristics of the products were different due to the different degrees of the Maillard reaction.

#### 3.2.2. Electronic Tongue Evaluation of Four Maillard Reaction Products

Electronic tongue is an intelligent detection system developed based on the mechanism of simulating human taste perception. This taste sensor is a potential type of sensor similar to the human taste system, which can quickly evaluate various basic taste indicators of food in digital form. The evaluation results of Ga-MRPs, Gl-MRPs, A-MRPs, and X-MRPs using the electronic tongue are shown in Table 1. Ga-MRPs has the highest salinity and umami value (24.01 and 9.14), followed by GI-MRPs (20.04 and 8.16), A-MRPs (16.38 and 6.61), and X-MRPs (15.58 and 6.50). These results indicate that the Maillard reaction products of hexose have better salinization and umami increasing effects than those of pentose. Therefore, Ga-MRPs have been selected for the subsequent study of salinization mechanisms.

#### 3.2.3. Correlation of Saltiness Response Values between Electronic Tongue and Sensory Saltiness Scores

To verify the reliability of salinity testing, the Pearson correlation coefficient between the saltiness value of electronic tongue and sensory saltiness scores was used, and the results are shown in Table 2. The Pearson correlation coefficient of saltiness value determined by electronic tongue and sensory was 0.909, indicating a very significant correlation between them. These results also indicated that there was a consistency between the electronic tongue and sensory evaluation in the evaluation of saltiness. The combination of electronic tongue and sensory evaluation is beneficial for obtaining more reliable results. Fu et al. [41] used the electronic tongue and sensory evaluation to analyze the taste characteristics of oyster MRPs, and found that they exhibited similar patterns. Therefore, the electronic tongue can be used to predict the sensory scores. The electronic tongue compensates for the weakness of sensory evaluators that are prone to fatigue, while sensory evaluation compensates for the weakness of electronic tongues that are not perceived by the human body. Therefore, the combination of the two can be used to better evaluate the intensity of saltiness.

### 3.3. Color Difference Analysis of PP, PPH and Ga-MRPs

The Maillard reaction process can be reflected by the color difference of the products. Table 3 showed the color difference values of PP, PPH, and Ga-MRPs. As the reaction proceeded, the L* values became smaller and the values of a* and b* tended to increase. Compared with PPH, the L* values of Ga-MRPs significantly decreased, while a* value significantly increased to 46.49 and 7.56. This is because there is a brown substance generated during the reaction, resulting in the decrease in the brightness of the product and the increase in the redness value. Gao et al. [42] compared the color difference of the products before and after hydrolysis of morel mushrooms, and similarly found that the L* value of the hydrolyzed product was significantly lower and the a* value was significantly higher compared to the unhydrolyzed sample.

### 3.4. Molecular Weight Analysis of Ga-MRPs

The molecular weight distribution can reflect the molecular weight changes of the MRPs due to peptide cross-linking, peptide degradation, and polymerization. The molecular weight distributions of Ga-MRPs are shown in Table 4. The molecular weights of Ga-MRPs are all below 3000 Da, and a large number of studies have demonstrated that small molecule proteins have the advantages of improving flavor, easy absorption, and increasing functional activity. The molecular weight of PPH below 500 Da determined in a previous study could reach 18.35%, which was higher than that of the Ga-MRPs [24]. These results were probably because the large molecular weight product was obtained through cross-linking of reducing sugars and peptide chains during the Maillard reaction process.

### 3.5. Amino Acid Analysis of Ga-MRPs

Amino acids play an important role in the flavor presentation of Maillard reaction products. By comparing the total and free amino acid contents of PPH and MRPs, the salinization mechanism of Maillard reaction was analyzed. Currently, research has shown that free amino acids and peptides have a significant impact on taste, and umami and sweet amino acids have a synergistic effect on increasing saltiness [43]. The total and free amino acid contents of Ga-MRPs are shown in Table 5. The contents of umami amino acids (glutamic, aspartic, glycine, and alanine) in Ga-MRPs were 9.1 mg/g, and the contents of sweet amino acids (threonine, serine, proline, glycine, and alanine) were 9.2 mg/g. In previous studies, we reported the total and free amino acid content of PPH [24]. Compared with PPH, the proportion of umami amino acids in Ga-MRPs increased from 19.81% to 26.07%, while the proportion of sweet amino acids in Ga-MRPs increased from 22.04% to 26.36%. In addition, the free amino acid content (62.6 mg/g) in PPH accounts for 7.34% of the total amino acid content (853 mg/g), indicating that the enzymatic hydrolysate is mainly composed of low relative molecular weight peptides, which provide important flavors [44]. The total and free amino acid content of Ga-MRPs decreased by 27.63% and 44.25%, respectively, compared to PPH, which may be related to the Strecker degradation of amino acids, peptide degradation, and the formation of Maillard peptides through sugar and amino acid cross-linking during the Maillard reaction process. In addition, the content of bitter amino acids in the products after the Maillard reaction significantly decreased, indicating that the proportion of bitter amino acids undergoing cross-linking during the Maillard reaction was greater than that formed by degradation. The decrease in bitter amino acids content was due to the participation of bitter amino acids in the formation of the MRPs; at the same time, the small peptides containing hydrophobic amino acids reacted with sugar molecules, which led to the structural change of enzyme degradation products and a decrease in surface hydrophobicity. Therefore, the bitterness was reduced by the Maillard reaction.

### 3.6. Antioxidant Properties of PPH and Ga-MRPs

The Maillard reaction is beneficial to human health, especially in improving the oxidation properties of products. The reducing power is one of the indicators reflecting the antioxidant properties of foods. In the presence of antioxidants, ferric chloride or ferricyanide is reduced to ferrous form [45]. Therefore, the reduction ability of samples can be indirectly compared by measuring the absorbance of its product Prussian blue at 700 nm, with a greater absorbance indicating greater reduction ability. PPH and Ga-MRPs have a certain reduction ability, which is positively correlated with concentration. When achieving the same reduction ability (at 0.5 absorbance), the concentration of PPH needs to be 30 g/L, while the concentration of Ga-MRPs only needs to be 0.5 g/L. It can be seen that compared with PPH, the reduction ability of Ga-MRPs was significantly enhanced.

**Figure 5:**
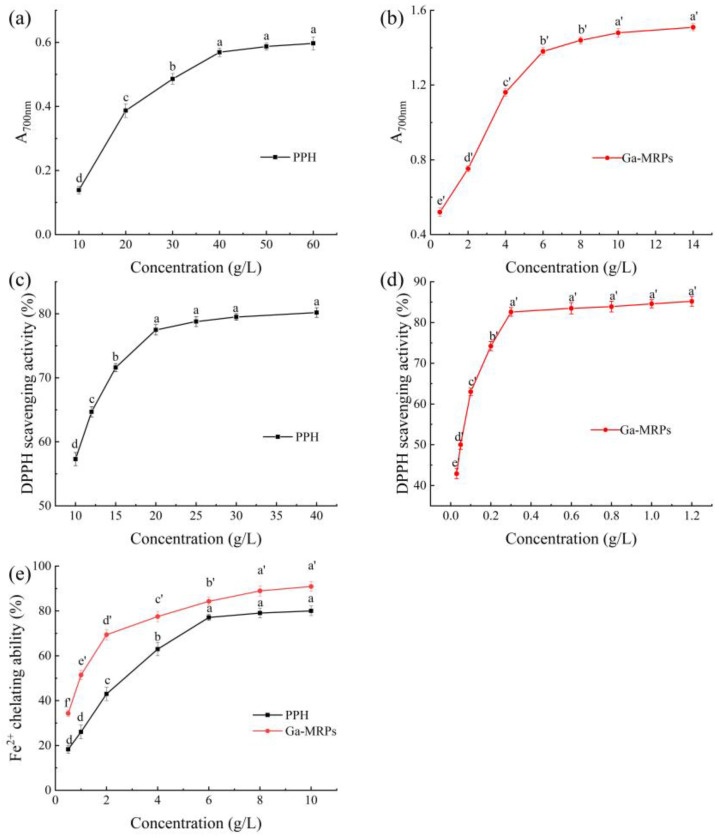
The antioxidant properties of PPH and Ga-MRPs. (**a**,**b**) Reducing power of PPH and Ga-MRPs; (**c**,**d**) DPPH radical scavenging capacity of PPH and Ga-MRPs; (**e**) Fe^2+^ chelating capacity of PPH and Ga-MRPs. Different lowercase letters (a–d and a’–f’) above the same line indicate that one-way ANOVA of the mean values differs significantly (*p* < 0.05).

DPPH is a compound containing a chromophore structure, and the antioxidant active substance scavenges DPPH radicals by providing hydroxyl groups into stable DPPH molecules and is accompanied by a color shift from purple to yellow, which can be used to measure the radical scavenging ability of products by determining the absorbance at 517 nm. From Figure 5c,d, it can be seen that both PPH and Ga-MRPs have a certain DPPH radical scavenging ability, and both of them increased with the increase of concentration. When the concentration of PPH was 40 g/L, its scavenging rate of DPPH radicals was 80.2%, while the scavenging rate of DPPH radicals by the sample could reach 83.5% when the concentration of Ga-MRPs was only 0.6 g/L. It can be seen that Ga-MRPs showed stronger DPPH radical scavenging ability compared with PPH.

The free radicals can be produced in the Fenton reaction only in the presence of transition metal ions, especially Fe^2+^ and Cu^2+^ [46]. The free radicals can be generated by the Fenton reaction. In addition, transition metal ions can promote lipid oxidation reactions, with Fe^2+^ having the strongest pro-oxidant effect. Compounds with Fe^2+^ chelating ability can reduce the concentration of Fe^2+^, thereby inhibiting lipid oxidation [47]. As can be seen in Figure 5e, the chelating ability of Fe^2+^ gradually increased with the increase of PPH’s and Ga-MRPs’ concentrations, and the growth rate of their chelation rates tended to level off when the concentration was greater than 30 g/L. At the same concentration of 10 g/L, the chelation rate of PPH was 80.05%, while that of Ga-MRPs was 90.93%. Therefore, Ga-MRPs had a higher Fe^2+^ chelating ability than PPH.

## 4. Discussion

The type of sugar affects the rate of the Maillard reaction. In this study, PPH was subjected to Maillard reactions with xylose, arabinose, glucose, and galactose, respectively, using the same reaction condition. It was found that the UV and fluorescence absorption spectra of pentose (xylose and arabinose) were higher than those of hexose (glucose and galactose), which was caused by the different structures of the sugars. The pentose, compared with hexose, has shorter carbon chains and smaller spatial steric hindrance of the carbon framework, which are more active and likely to penetrate deeper into the folded structure of the peptide chain to react with amino groups. During the Maillard reaction, a condensation reaction occurs between the carbonyl group of the reducing sugar and the amino group of proteins to form Schiff bases, which are then converted to the Amadori rearrangement products at a fast rate. When Shang et al. [48] investigated the effect of sugar types (xylose, ribose, glucose, fructose, and galactose) on the structure of Maillard reaction products for peony seed meal, it was found that the higher the degree of the Maillard reaction, the easier it was to promote the conversion of small molecular weight peptides into mellow-flavored glycopeptide cross-linking products through the Maillard reaction. Moreover, the concentration of umami-flavored small peptides with an increasing saltiness effect decreased, resulting in a weakened salt effect. The saltiness of the pea peptide hexose (glucose and galactose) system was significantly higher than that of its pentose (xylose, ribose, and fructose) system. Yan et al. [49] found that pentasaccharides were more active than hexasaccharides in the Maillard reaction, and the Maillard reaction products of hexasaccharides had a higher umami enhancement using electron tongue analysis of the pea peptides MRPs with different sugar sources. However, their study also found that only Gl-MRPs had a saltiness-enhancing effect, while MRPs prepared from xylose, arabinose, ribose, and galactose did not have the effect of increasing saltiness. This finding is different from the results of the present study, which may be due to the fact that the precursor of the Maillard reaction in this study was peanut protease hydrolysate. Compared to pea protein, peanut protein has a higher content of arginine; it is possible that different salinization products were generated during the Maillard reaction process. Previous researchers have found that the dipeptide Ala-Arg significantly increased the response of amiloride-sensitive receptors ENaC-α and ENaC-δ, resulting in an enhanced saltiness [50]. The molecular of 500–3000 Da in MRPs is higher than that of PPH, which may be due to the higher activity of amino acids and the fact that N-terminal amino acids of low-molecular polypeptides in the Maillard reaction can easily cause polymerization and cross-linking reactions, which cross-links the peptide chains or their degradation products by reducing sugars to obtain products with large molecular weight.

The composition and content of amino acids are important indicators of nutritional quality and sensory performance of foods. Compared with the amino acids of PPH in previous studies, the amino acid content of Ga-MRPs was reduced with varying degrees after the Maillard reaction, which was associated with Strecker degradation and thermal degradation of amino acids as well as the cross-linking of free amino acids and reducing sugars during the Maillard reaction. It has been shown that hydrophobic amino acids such as Val, Ile, Leu, Tyr, Phe, and Lys released bitterness. Comparing the amino acid content of this study with PPH in previous studies, it was found that the bitter amino acid content of Ga-MRPs was decreased, which indicated that bitter amino acids were involved in the formation of Maillard reaction products. It may be that small peptides containing hydrophobic amino acids react with the sugar molecules, causing changes in the structure of peanut peptides, covering up the exposure of bitter amino acids. Therefore, the bitterness of the PPH was reduced through the Maillard reaction. Both sensory and electronic tongue evaluations showed that the salty and umami of the products were increased after the Maillard reaction; and although the umami amino acid content of the products decreased after the reaction, the proportion of umami amino acids to the total free amino acids increased. On the other hand, the introduction of sugar groups during the Maillard reaction may also be the reason for the increased saltiness and umami of the product [37].

Maillard reactions improve the scavenging ability of DPPH radicals of PPH, probably because of the production of a large number of volatile sulfur-, nitrogen-, and oxygen-containing heterocyclic compounds during the Maillard reaction process. The uneven distribution of π electrons in the heterocyclic compounds on the ring results in excess electrons on the carbon atom and an increase in π electron cloud density, and promotes the electrophilic addition of free radicals, thereby exhibiting strong scavenging ability [51]. The chelating ability of the samples to chelate iron ions can be significantly improved by the Maillard reaction, and it may also be related to the composition of the amino acids in the samples. It has been reported that histidine has a strong chelating ability due to the imidazole ring on its residues being able to form polymers with ferric ions. Some acidic amino acids (glutamic acid and aspartic acid) or basic amino acids (lysine, arginine, and histidine) also play an important role in chelating ferric ions [52]. Qiu et al. [53] investigated the DPPH, ABTS, and hydroxyl radical scavenging capacity, and reducing capacity, of mushroom hydrolysates before and after the Maillard reaction. They found that the DPPH and ABTS radical scavenging capacity and reducing capacity of the products after the Maillard reaction were increased, while the hydroxyl radical scavenging capacity was decreased.

## 5. Conclusions

Taken together, the Maillard reaction between hexose (Gl-MRPs and Ga-MRPs) and peanut protein hydrolysate is not as strong as that of pentose (X-MRPs and A-MRPs) under the same conditions, but their Maillard product has higher saltiness and freshness enhancement effects than those of the pentose Maillard product. The Maillard reaction can improve the flavor of PPH. Compared with PPH, the content of amino acids and free amino acids in the MRPs decreased, and the content of small peptides with molecular weights of 500~3000 Da increased, and showed stronger salty and umami effects, which was attributed to the fact that the amino acids and the low molecular peptides reacted through cross-linking during the Maillard reaction to produce macromolecular products with increasing saltiness and umami properties. In addition, the content of bitter amino acids in Ga-MRPs was reduced compared to PPH. The content of umami amino acids in Ga-MRPs was reduced, but the proportion of umami amino acids in the total free amino acids was increased. Sensory and electronic tongue evaluations have shown that the saltiness and umami of the hydrolysate after the Maillard reactions have been improved. In addition, the Ga-MRPs had an enhanced reducing ability, DPPH radical scavenging ability, and Fe^2+^ chelating ability compared with PPH. This study provides a theoretical basis for the use of peanut protein Maillard products as a salt-reducing agent and a food antioxidant.

## Figures and Tables

**Table 1:** Electron tongue evaluation of four Maillard reaction products.

Sugar Type	Sourness	Bitterness	Astringency	Umami	Richness	Saltiness
X-MRPs	−12.61 ± 0.01 ^a^	1.56 ± 0.01 ^a^	−0.75 ± 0.01 ^a^	6.50 ± 0.03 ^d^	1.35 ± 0.03 ^d^	15.58 ± 0.02 ^d^
A-MRPs	−14.37 ± 0.01 ^b^	1.50 ± 0.10 ^b^	−5.85 ± 0.02 ^b^	6.61 ± 0.01 ^c^	1.79 ± 0.02 ^c^	16.38 ± 0.03 ^c^
Gl-MRPs	−17.73 ± 0.02 ^c^	0.72 ± 0.12 ^c^	−9.75 ± 0.01 ^c^	8.16 ± 0.05 ^b^	1.97 ± 0.01 ^b^	20.04 ± 0.04 ^b^
Ga-MRPs	−20.17 ± 0.01 ^d^	0.50 ± 0.12 ^d^	−12.59 ± 0.01 ^d^	9.14 ± 0.03 ^a^	2.25 ± 0.02 ^a^	24.01 ± 0.01 ^a^

Different lowercase letters after the means as the superscripts in the same column indicate that one-way ANOVA of the means differs significantly (*p* < 0.05).

**Table 2:** Pearson correlation analysis of electronic tongue and sensory saltiness evaluation.

		Electronic Tongue Saltiness Value	Sensory Saltiness Value
Electronic tongue saltiness value	Pearson Correlation	1	0.909 **
	Sig.		0.000
	Number of cases	20	20
Sensory saltiness value	Pearson Correlation	0.909 **	1
	Sig.	0.000	
	Number of cases	20	20

** indicates a highly significant difference (*p* < 0.01).

**Table 3:** The color change of products before and after the Maillard reaction.

Samples	L*	a*	b*
PP	52.48 ± 0.05 ^a^	6.96 ± 0.12 ^c^	7.51 ± 0.12 ^c^
PPH	47.94 ± 0.34 ^b^	7.34 ± 0.13 ^b^	8.59 ± 0.17 ^b^
Ga-MRPs	46.49 ± 0.31 ^c^	7.56 ± 0.16 ^a^	9.61 ± 0.15 ^a^

Different lowercase letters after the means as the superscripts in the same column indicate that one-way ANOVA of the means differs significantly (*p* < 0.05).

**Table 4:** The molecular weight distribution of the Ga-MRPs.

Molecular Weight Range (Da)	Ga-MRPs (%)
<100	1.82 ± 0.06
100~500	15.4 ± 0.97
500~1000	46.7 ± 1.76
1000~3000	36.08 ± 2.01

**Table 5:** The total and free amino acid contents of Ga-MRPs.

Amino	Total Amino Acids (mg/g)	Free Amino Acids (mg/g)
Asp	41 ± 0.37	0.9 ± 0.00
Thr	13.4 ± 0.11	1.5 ± 0.00
Ser	22.5 ± 0.21	1.7 ± 0.01
Glu	79.7 ± 0.41	2.2 ± 0.02
Gly	160 ± 0.86	2.9 ± 0.01
Ala	63.0 ± 0.47	3.1 ± 0.10
Val	17.7 ± 0.16	1.1 ± 0.00
Met	7.7 ± 0.09	-
Ile	10.9 ± 0.1	1.1 ± 0.01
Leu	22.9 ± 0.22	4.3 ± 0.02
Tyr	3.6 ± 0.07	-
Phe	14.0 ± 0.18	5.6 ± 0.06
His	4.3 ± 0.09	2.6 ± 0.05
Arg	53.9 ± 0.44	5.3 ± 0.01
Pro	79.3 ± 0.38	-
Lys	23.4 ± 0.05	2.6 ± 0.12
Essential amino acid content	110 ± 1.02	16.2 ± 0.16
Umami amino acids	198 ± 1.23	9.1 ± 0.13
Sweet amino acids	338.2 ± 1.37	9.2 ± 0.12
Bitter amino acids	92.5 ± 1.21	14.7 ± 0.27
Total amount of amino acids	617.3 ± 1.87	34.9 ± 0.31

“-” indicates not detected.

## Data Availability

The original contributions presented in the study are included in the article, further inquiries can be directed to the corresponding authors.

## Abbreviations

PP	peanut protein
PPH	peanut protein hydrolysates
MRPs	Maillard reaction products
Ga-MRPs	galactose-Maillard reaction products
Gl-MRPs	glucose-Maillard reaction products
X-MRPs	xylose-Maillard reaction products
A-MRPs	arabinose-Maillard reaction products

## References

[1] Hariharan S., Patti A., Arora A. (2023). Functional proteins from biovalorization of peanut meal: Advances in process technology and applications. Plant Foods Hum. Nutr..

[2] Shi A., Liu H., Liu L., Hu H., Wang Q., Adhikari A. (2014). Isolation, purification and molecular mechanism of a peanut protein-derived ACE-inhibitory peptide. PLoS ONE.

[3] Ruffin E., Schmit T., Lafitte G., Dollat J., Chambin O. (2014). The impact of whey protein preheating on the properties of emulsion gel bead. Food Chem..

[4] Sangtanoo P., Srimongkol P., Saisavoey T., Reamtong O., Karnchanatat A. (2020). Anti-inflammatory action of two novel peptides derived from peanut worms (*Sipunculus nudus*) in lipopolysaccharide-induced RAW264.7 macrophages. Food Funct..

[5] Zhao F., Zhai X., Liu X., Lian M., Liang G.T., Cui J.X., Dong H.Z., Wang W.T. (2021). Effects of High-Intensity ultrasound pretreatment on structure, properties, and enzymolysis of walnut protein isolate. Molecules.

[6] Li C., Zhu B., Xue H.R., Chen Z.Y., Ding Q., Wang X.G. (2013). Physicochemical properties of dry-heated peanut protein isolate conjugated with dextran or gum arabic. Am. Oil Chem. Soc..

[7] Jamdar S.N., Rajalakshmi V., Pednekar M.D., Juan F., Yardi V., Sharma A. (2010). Influence of degree of hydrolysis on functional properties, antioxidant activity and ACE inhibitory activity of peanut protein hydrolysate. Food Chem..

[8] Zhang J., Sun-Waterhouse D., Feng Y., Su G., Zhao M., Lin L. (2018). The umami intensity enhancement of peanut protein isolate hydrolysate and its derived factions and peptides by Maillard reaction and the analysis of peptide (EP) Maillard products. Food Res. Int..

[9] Deckers I., Van D., Engeland M.V., Soetekouw P.M.M.B., Baldewijns M.M.L.L., Goldbohm R.A., Schouten L.A. (2014). Long-term dietary sodium, potassium and fluid intake; exploring potential novel risk factors for renal cell cancer in the Netherlands cohort study on diet and cancer. Br. J. Cancer.

[10] Wilck N., Matus M.G., Kearney S.M., Olesen S.W., Forslund K., Bartolomaeus H., Haase S., Mähler A., Balogh A., Markó L. (2017). Salt-responsive gut commensal modulates TH 17 axis and disease. Nature.

[11] Garofalo C., Borrelli S., Provenzano M., Stefano T.D., Vita C., Chiodini P., Minutolo R., Nicola L., Conte G. (2018). Dietary salt restriction in chronic kidney disease: A meta-analysis of randomized clinical trials. Nutrients.

[12] Nasri N., Septier C., Beno N., Salles C., Thomas-Danguin T. (2013). Enhancing salty taste through odour–taste–taste interactions: Influence of odour intensity and salty tastants’ nature. Food Qual. Prefer..

[13] Dugat-Bony E., Bonnarme P., Fraud S., Catellote J., Sarthou A., Loux V., Rué O., Bel N., Chuzeville S., Helinck S. (2019). Effect of sodium chloride reduction or partial substitution with potassium chloride on the microbiological, biochemical and sensory characteristics of semi-hard and soft cheeses. Food Res. Int..

[14] Tada M., Shinoda I., Okai H. (1984). L-ornithyltaurine, a new salty peptide. Agric. Food Chem..

[15] Zhang C., Alashi A.M., Singh N., Chelikani P., Aluko R.E. (2019). Glycated beef protein hydrolysates as sources of bitter taste modifiers. Nutrients.

[16] Liu L.L., Li Y., Prakash S., Dai X.N., Meng Y.Y. (2018). Enzymolysis and glycosylation synergistic modified ovalbumin: Functional and structural characteristics. Int. J. Food Prop..

[17] Feng T., Zhou Y., Wang X., Wang X., Xia S. (2021). α-Dicarbonyl compounds related to antimicrobial and antioxidant activity of maillard reaction products derived from xylose, cysteine and corn peptide hydrolysate. Food Biosci..

[18] Zhang W.W., Han Y.Q., Shi K.X., Wang J.M., Yang C., Xu X. (2022). Effect of different sulfur-containing compounds on the structure, sensory properties and antioxidant activities of Maillard reaction products obtained from *Pleurotus citrinopileatus* hydrolysates. LWT.

[19] Shakoor A., Zhang C.P., Xie J.C., Yang X.L. (2022). Maillard reaction chemistry in formation of critical intermediates and flavour compounds and their antioxidant properties. Food Chem..

[20] Ni Z.J., Liu X., Xia B., Hu L.T., Thakur K., Wei Z.J. (2021). Effects of sugars on the flavor and antioxidant properties of the Maillard reaction products of camellia seed meals. Food Chem. X.

[21] Naik R.R., Ye Q.Y., Wang Y., Selomulya C. (2024). Assessing the effect of Maillard reaction products on the functionality and antioxidant properties of Amaranth-red seaweed blends. Food Res. Int..

[22] Viturat S., Thongngam M., Lumdubwong N., Zhou W.B., Klinkesorn U. (2023). Ultrasound-assisted formation of chitosan-glucose Maillard reaction products to fabricate nanoparticles with enhanced antioxidant activity. Ultrason. Sonochem..

[23] Han J.R., Yan J.R., Sun S.G., Tang Y., Shang W.H., Li A.T., Guo X.K., Du Y.N., Wu H.T., Zhu B.W. (2018). Characteristic antioxidant activity and comprehensive flavor compound profile of scallop (*Chlamys farreri*) mantle hydrolysates-ribose Maillard reaction products. Food Chem..

[24] Yang C.H., Xing W.J., Chen F.L., Liang M.L., Zhang N. (2023). Process optimization for the preparation of salt-enhancing digests based on deep enzymatic digestion of peanut proteins. J. Food Saf. Qual..

[25] Sun L.B., Wang D.H., Huang Z., Elfalleh W., Qin L.X., Yu D.Y. (2023). Structure and flavor characteristics of Maillard reaction products derived from soybean meal hydrolysates-reducing sugars. LWT.

[26] Wang W., Zhang L., Wang Z., Wang X., Liu Y. (2019). Physicochemical and sensory variables of Maillard reaction products obtained from *Takifugu obscurus* muscle hydrolysates. Food Chem..

[27] Liu L.U., Li X., Du L., Zhang X., Yang W., Zhang H. (2019). Effect of ultrasound assisted heating on structure and antioxidant activity of whey protein peptide grafted with galactose. LWT Food Sci. Technol..

[28] Liu Y., Zhao G., Zhao M., Ren J., Yang B. (2012). Improvement of functional properties of peanut protein isolate by conjugation with dextran through Maillard reaction. Food Chem..

[29] He S.D., Zhang Z.Y., Sun H.J., Zhu Y.C., Zhao J.L., Tang M.M., Wu X.Y., Cao Y.P. (2019). Contributions of temperature and L-cysteine on the physicochemical properties and sensory characteristics of rapeseed flavor enhancer obtained from the rapeseed peptide and D-xylose Maillard reaction system. Ind. Crops Prod..

[30] Yu B.B., Wu W., Wang B., Zhang N., Bak K.H., Soladoye O.P., Aluko R.E., Zhang Y.H., Fu Y. (2022). Maillard-reacted peptides from glucosamine-induced glycation exhibit a pronounced salt taste-enhancing effect. Food Chem..

[31] Zha F.C., Dong S.Y., Rao J.J. (2019). The structural modification of pea protein concentrate with gum Arabic by controlled Maillard reaction enhances its functional properties and flavor attributes. Food Hydrocoll..

[32] Wu Q., Cai Q.F., Tao Z.P., Sun L.C., Shen J.D., Zhang L.J., Liu G.M., Cao M.J. (2015). Purification and characterization of a novel angiotensin I-converting enzyme inhibitory peptide derived from abalone (*Haliotis discus* hannai Ino) gonads. Eur. Food Res. Technol..

[33] Zhao T.T., Zhang Q., Wang S.G., Qiu C.Y., Liu Y., Su G.W., Zhao M.M. (2018). Effects of Maillard reaction on bioactivities promotion of anchovy protein hydrolysate: The key role of MRPs and newly formed peptides with basic and aromatic amino acids. LWT–Food Sci. Technol..

[34] Sampath K.N.S., Nazeer R.A., Jaiganesh R. (2012). Purification and identification of antioxidant peptides from the skin protein hydrolysate of two marine fishes, horse mackerel (*Magalaspis cordyla*) and croaker (*Otolithes ruber*). Amino Acids.

[35] Liu H.M., Chen J., Hao L.W., Yu H. (2018). Antioxidant activity of maillard reaction products from glucose and oyster enzymatic hydrolysate. Food Sci..

[36] Pino F.R., Galvez R., Carpio F., Guadix E. (2020). Evaluation of Tenebrio molitor protein as a source of peptides for modulating physiological processes. Food Funct..

[37] Fu Y., Liu J., Zhang W., Wæhrens S.S., Tøstesen M., Hansen E.T., Lametsch R. (2020). Exopeptidase treatment combined with Maillard reaction modification of protein hydrolysates derived from porcine muscle and plasma: Structure–taste relationship. Food Chem..

[38] Chen X., Jiang D., Xu P., Geng Z., Xu W. (2021). Structural and antimicrobial properties of Maillard reaction products in chicken liver protein hydrolysate after sonication. Food Chem..

[39] Chen W.J., Ma X.B., Wang W.J., Lv R.L., Guo M.M., Ding T., Ye X.Q., Miao S., Liu D.H. (2019). Preparation of modified whey protein isolate with gum acacia by ultrasound Maillard reaction. Food Hydrocoll..

[40] Liu L.P., Lu X.M., Li N.Y., Zheng Z.J., Qiao X.G. (2019). Characterization, variables, and antioxidant activity of the Maillard reaction in a fructose-histidine model system. Molecules.

[41] Fu B.F., Xu X.B., Zhang X., Cheng S.Z., ElSeedi H.R., Du M. (2023). Identification and characterisation of taste-enhancing peptides from oysters (*Crassostrea gigas*) via the Maillard reaction. Food Chem..

[42] Gao J., Fang D.L., Muinde K.B., Chen X., Wu X., Du J.X., Yang Q., Chen H., Zheng H.H., An X.X. (2021). Analysis of umami taste substances of morel mushroom (*Morchella sextelata*) hydrolysates derived from different enzymatic systems. Food Chem..

[43] Zheng Y., Tang L., Yu M., Li T., Song H.L., Li P., Li K., Xiong J. (2020). Fractionation and identification of salty peptides from yeast extract. J. Food Sci. Technol..

[44] Kong Y., Yang X., Ding Q., Zhang Y.Y., Sun B.G., Chen H.T., Sun Y. (2017). Comparison of non-volatile umami components in chicken soup and chicken enzymatic hydrolysate. Food Res. Int..

[45] Vhangani L.N., Wyk J.V. (2013). Antioxidant activity of Maillard reaction products (MRPs) derived from fructose-lysine and ribose-lysine model systems. Food Chem..

[46] Nooshkam M., Varidi M., Bashash M. (2019). The Maillard reaction products as food-born antioxidant and antibrowning agents in model and real food systems. Food Chem..

[47] Bai W.D., Wang Q., Zeng X.F., Fu J.Y., Liu Y.R., Dong H. (2017). Antioxidant activities of chicken peptide-Maillard reaction products (Cp-Mrps) derived from chicken peptides and D-glucose system. J. Food Process. Preserv..

[48] Shang Y.F., Cao H., Wei C.K., Thakur K., Liao A.M., Huang J.H., Wei Z.J. (2020). Effect of sugar types on structural and flavor properties of peony seed derived Maillard reaction products. J. Food Process. Preserv..

[49] Yan F., Cui H.P., Zhang Q., Hayat K., Yu J.Y., Hussain S., Tahir M.U., Zhang X.M., Ho C.T. (2021). Small peptides hydrolyzed from pea protein and their Maillard reaction products as taste modifiers: Saltiness, Umami, and Kokumi enhancement. Food Bioprocess Technol..

[50] Xu J.J., Elkaddi N., Garcia-Blanco A., Spielman A.I., Bachmanov A.A., Chung H.Y., Ozdener M.H. (2017). Arginyl dipeptides increase the frequency of NaCl-elicited responses via epithelial sodium channel alpha and delta subunits in cultured human fungiform taste papillae cells. Sci. Rep..

[51] Huang M., Liu P., Song S., Zhang X.M., Hayat K., Xia S.Q., Jia C.S., Gu F.L. (2011). Contribution of sulfur-containing compounds to the colour-inhibiting effect and improved antioxidant activity of Maillard reaction products of soybean protein hydrolysates. J. Sci. Food Agric..

[52] Lancker F.V., Adams A., Kimpe N.D. (2010). Formation of pyrazines in Maillard model systems of lysine-containing dipeptides. J. Agric. Food Chem..

[53] Qiu J.G., Li H.Y., Liu Y., Li C., Fang Z.F., Hu B., Li X.L., Zeng Z., Liu Y.T. (2023). Changes in flavor and biological activities of Lentinula edodes hydrolysates after Maillard reaction. Food Chem..

